# Comparison of Analgesic Efficacy between Single Interscalene Block Combined with a Continuous Intra-bursal Infusion of Ropivacaine and Continuous Interscalene Block after Arthroscopic Rotator Cuff Repair

**DOI:** 10.4055/cios.2009.1.1.48

**Published:** 2009-02-06

**Authors:** Joo Han Oh, Ka-Young Rhee, Sae Hoon Kim, Pyung-Bok Lee, Joon-Woo Lee, Seok Jae Lee

**Affiliations:** Department of Orthopaedic Surgery, Seoul National University College of Medicine, Korea.; * Department of Anesthesiology and Pain Medicine, Konkuk University School of Medicine, Korea.; † Department of Anesthesiology and Pain Medicine, Seoul National University College of Medicine, Seoul, Korea.; ‡ Department of Radiology, Seoul National University College of Medicine, Seoul, Korea.

**Keywords:** Shoulder, Rotator cuff tear, Postoperative pain control, Continuous interscalene block, Single interscalene block with intra-bursal infusion, Arthroscopic rotator cuff repair

## Abstract

**Background:**

This study evaluated the effectiveness of a continuous interscalene block (CISB) by comparing it with that of a single interscalene block combined with a continuous intra-bursal infusion of ropivacaine (ISB-IB) after arthroscopic rotator cuff repair.

**Methods:**

Patients who had undergone CISB (CISB group; n = 25) were compared with those who had undergone ISB-IB (ISB-IB group; n = 25) for more than 48 hours after surgery. The visual analog scale (VAS) for pain, motor and/or sensory deficit, supplementary analgesics and adverse effects were recorded.

**Results:**

There were no significant differences between the postoperative VAS of the CISB and ISB-IB groups, except at 1 hour after surgery. Their supplementary analgesics of the two groups were similar. Transient motor weakness (52%) and sensory disturbance (40%) of the affected arm were observed in patients in the CISB group. The catheters came out accidentally in 22% of the CISB group but in only 4% of the ISB-IB group.

**Conclusions:**

ISB-IB provides similar analgesia to CISB. However, the ISB-IB group had a lower incidence of neurological deficits and better catheter retention.

Appropriate pain control after shoulder surgery has been reported to enhance postoperative rehabilitation, early mobilization and functional recovery, including the range of motion (ROM) and muscle power. It also helps prevent serious complications, such as deep vein thrombosis (DVT) and osteoporosis, as well as shorten the hospital stay. Therefore, aggressive pain management in the early postoperative period is a major issue for patients who pursue "well-being" and satisfaction with the treatment of their disorders.

Recently, an interscalene block was demonstrated to provide successful anesthesia for shoulder surgery, and postoperative continuous interscalene analgesia is considered to be quite effective after a variety of shoulder surgical procedures.^1^,2) However, a continuous peripheral nerve block can ultimately fail leading to serious complications during the infusion period. We previously examined whether a single interscalene block with a continuous infusion of intra-lesional ropivacaine could effectively relieve postoperative pain, and reported fewer side effects after arthroscopic shoulder surgery compared with a continuous infusion of intravenous (IV) opioids.3)

Therefore, this prospective study compared the efficacy in postoperative pain control of a continuous interscalene block with that of a combination of single interscalene block with a continuous intra-bursal infusion of local anesthetics after arthroscopic rotator cuff repair. It was hypothesized that a combination of a single interscalene block with a continuous intra-bursal infusion of ropivacaine would be a good alternative to a continuous interscalene block.

## METHODS

This study was a prospective randomized case-control study. After obtaining approval from the Institutional Review Board of the first author's institution and written informed consent from all patients, 58 consecutive adults were enrolled for a scheduled elective unilateral arthroscopic rotator cuff repair. All patients met the American Society of Anesthesiologists criteria of physical status I or II. The exclusion criteria included the following: patients with massive cuff tears, open or miniopen rotator cuff repair, partial repair, cardiac (ischemic heart disease, arrhythmia) or respiratory (SpO_2_ < 95%) disorders, pregnancy, moderate to severe obesity (body mass index > 30), an age exceeding 70 years, preexisting neuropathy, coagulopathy, and/or allergy to local anesthetics.

The concentration of local anesthetics (ropivacaine) used in the continuous interscalene block (CISB) group was determined from a pilot study with sixteen patients. The optimal concentration was determined to cause complete sensory block with recovery occurring within 2 hours of the end of infusion. The concentrations and infusion rates of ropivacaine administered to the single interscalene block combined with continuous intra-bursal infusion (ISB-IB) group were the same as those reported previously.^3^-5)

The 58 patients were allocated randomly to the CISB and ISB-IB groups using a table of random sampling numbers. In the CISB group (n = 32), a perineural catheter was inserted via the interscalene or parascalene approach with ultrasound (Envisor, Philips Medical Systems, Eindhoven, Netherlands) guidance. An interscalene block was performed through a perineural catheter using 15 ml of 0.15% ropivacaine prior to surgery. The success of the block was evaluated by the presence of a sensory block after 30 minutes. At the end of surgery, the location of the catheter tip was confirmed using the contrast media and fluoroscopy. The catheter was fixed firmly with adhesive tape. Subsequently, 0.15% ropivacaine was infused via a PCRA (patient-controlled regional analgesia) system (Automed 3200, Ace Medical, Seoul, Korea) for the first 48 hours after surgery. The basal infusion rate, bolus and lockout interval were 4 ml/hr, 2 ml and 20 min, respectively. In the ISB-IB group (n = 26), a single-injection interscalene block was performed using 20 ml of 0.25% ropivacaine via the interscalene approach using a 50 mm, 22 gauge short-beveled needle (Stimuplex, B Braun, Melsungen, Germany) connected to a nerve stimulator (Stimuplex- DIG, B Braun, Melsungen, Germany) prior to surgery. The success of the block was assessed by the observance of sensory loss after 30 minutes. At the end of surgery, a catheter was inserted into the subacromial space through an anterosuperior portal in the sterile operation field, and tagged firmly with nylon. After aspirating the remaining fluid from the subacromial space, a 10 ml bolus of 0.75% ropivacaine was injected, followed by the continuous infusion of 0.5% ropivacaine at a rate of 2 ml/hr for 48 hours postoperatively.

All patients received general anesthesia. Induction was achieved with propofol 1.5 to 2 mg/kg, rocuronium 0.6 to 1.0 mg/kg, and fentanyl 1 to 2 µg/kg, and general anesthesia was maintained with 2 to 5% sevoflurane and 66% N_2_O in oxygen. Arthroscopic subacromial decompression and acromioplasty was performed in all patients. The arthroscopic cuff repair was performed with suture anchors. A single row technique was used for small tears, and a double row technique was used for medium to large-sized tears. Ten patients in the CISB group and 11 in ISB-IB had combined lesions (biceps partial tear, AC arthritis, SLAP lesion, etc.), which were fixed according to their pathology.

The postoperative resting pain was assessed using the visual analogue scale (VAS) at 1 hour after surgery and then at every 8 hours for 48 hours. If the VAS exceeded 60 mm, i.e., the median value for moderate pain,6) or if there were requests from patients for further pain control, ketorolac (30 mg) and then meperidine (50 mg) were administered intravenously in that order. Sensory loss of the affected upper extremity was expressed as the percentage deficit versus the normal side, and motor function was evaluated using a 5-point scoring system for muscle power regarding wrist flexion, wrist extension, finger abduction and finger flexion. Abnormal symptoms, such as tingling, perioral numbness, hearing disturbance, visual disturbance, dysarthria, dizziness, nausea and vomiting, were recorded during the first 48 hours after surgery.

Shoulder shrugging and active motions of the fingers, wrist and elbow were permitted immediately after the procedure. Passive shoulder motion was started from the day after surgery, and a pain assessment was performed at rest or after at least one hour of therapeutic exercise.

Statistical analyses were carried out by a statistical consultant (Medical Research Collaborating Center, Seoul National University College of Medicine, Seoul, Korea). Twenty-three patients in each group were required to demonstrate a 20% difference between the groups in VAS at an α level of 0.05 and a β value of 0.10 according to power analysis. Statistical analyses were carried out using SPSS ver. 12.0. The patient's age, preoperative/postoperative VAS values, and surgery time were analyzed using an unpaired two-tailed t-test. Chi-square analysis was used to compare the two groups with respect to gender, concomitant diseases, ASA physical status, the number of patients taking supplementary analgesics, and the incidence of side effects. A *p* < 0.05 was considered significant.

## RESULTS

The catheters came out accidentally in 22% (7/32) of patients in the CISB group but in only 4% (1/26) of patients in the ISB-IB group. Their pain control was changed to an intravenous opioids infusion using a PCA manner. These patients were excluded from statistical analysis. Overall, 25 patients in each group complied with the study protocol. The demographic data and preoperative variables of the two groups, including size of the tear, preoperative pain VAS and operation time, were statistically similar (Table 1).

**Fig. 1:**
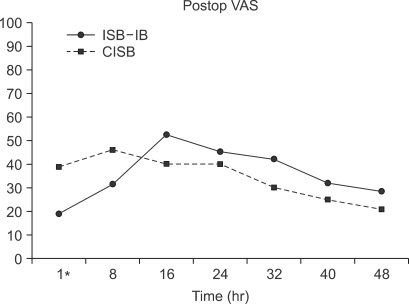
Postoperative pain VAS showing a similar level of pain in the CISB and ISB-IB group, except at 1 hour after surgery. ^*^At this time, the pain VAS was higher in the CISB group than in the ISB-IB group (*p* < 0.05).

**Fig. 2:**
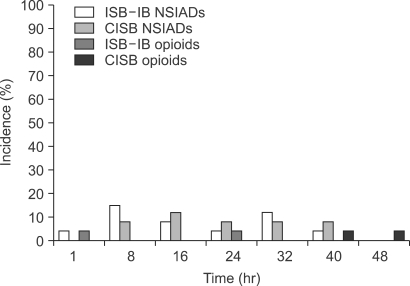
The additional analgesic requirements were similar in both groups.

The level of postoperative pain VAS was similar in the two groups, except at 1 hour after surgery. The pain VAS of the CISB group was significantly higher than that of the ISB-IB group (Fig. 1). Additional analgesic intake did not result in any significant difference between the two groups (Fig. 2).

Slightly more than half of the patients (52%) in the CISB group complained of motor weakness in the upper extremities during continuous infusion. Ten patients (40%) in the CISB group complained of persistent sensory disturbance for more than 2 hours after completing the infusion. A tingling sensation remained for one day in three patients, and for three weeks in one patient. In the CISB group, nausea developed in two patients (5%), and ptosis was encountered in one patient (3%). However, there were no remarkable complications in the patients of the ISB-IB group.

## DISCUSSION

A previous study compared the effectiveness of postoperative pain control via the continuous intralesional infusion of ropivacaine with IV PCA of opioid, with and without an interscalene block after arthroscopic shoulder surgery.3) In that study, a single-dose interscalene block with a continuous intralesional infusion of ropivacaine provided effective pain control and reduced the supplementary analgesic requirement with few side effects and high patient satisfaction. Klein et al.5) also reported that the continuous intra-articular infusion of ropivacaine combined with a single-dose interscalene block provided better pain relief than with an interscalene block alone. Although the use of intrabursal analgesia is still controversial, the infiltration of local anesthetics or opioids into the subacromial space has been reported to be effective.^4^,^5^,^7^-11) Moreover, the additional intrabursal infusion of local anesthetics to ISB would have positive effects on postoperative pain control because the initial benefits of ISB have a short duration.^8^,12)

On the other hand, CISB has gained increasing acceptance recently as a method of pain control in upper extremity surgery. Since CISB was introduced by Tuo-minen et al.13) and Borgeat et al.,^2^,14) other studies^15^,16) reported that CISB provides better postoperative analgesia than a continuous subacromial infusion or IV PCA. Both techniques, CISB and ISB-IB, effectively controlled the postoperative pain but no comparisons of the two methods have been reported. Therefore, a prospective randomized study was designed to compare CISB with ISB-IB after arthroscopic rotator cuff repair.

A continuous interscalene block is a common method of pain control after shoulder surgery. It can eliminate the need for general anesthesia in high risk patients, and reduce the level of intraoperative pain help maintain a low systolic blood pressure. Furthermore, CISB makes outpatient surgery possible, particularly arthroscopic shoulder surgery. However, CISB can be associated with dangerous complications, as well as poor patient compliance. Moreover, cooperation with the Department of Anesthesiology is essential for this time-consuming regional block. This prospective randomized controlled trial suggests that postoperative pain control by ISB-IB is as effective as CISB. However, ISB-IB resulted in a lower incidence of motor and sensory deficits as well as better catheter retention. Therefore, it is believed that ISB-IB is an effective and safe alternative for pain control after arthroscopic rotator cuff repair.

These results showed that the pain VAS was higher in the CISB group at 1 hour after surgery, which might be due to the smaller amount and lower concentration of ropivacaine used in the initial dose in the CISB group than in the ISB-IB group. This difference was attributed to the study design. The dose and concentration of ropivacaine used in the CISB group (15 ml, 0.15%) was determined by a pilot study, which titrated ropivacaine to the minimum concentration to achieve complete sensory block after 30 minutes, and recovery from the sensory block within 2 hours after completion of the continuous infusion. In the ISB-IB group, the regimen of the ropivacaine concentration (20 ml, 0.25%) was similar to that reported previously3) in order to allow a continuation of that study. It is possible that the higher pain VAS in the CISB group might be reduced if the initial dose of ropivacaine was the same.

"Rebound pain" means a sudden increase in pain 8 hours after surgery due to the reduced effect of ISB. This type of pain developed in the patients in the ISB-IB group, and is a common problem with this analgesic strategy. It is expected that "rebound pain" might be overcome with CISB by prolonging the effect of ISB in this critical period but the level of pain was higher than expected in our patients. The dose of ropivacaine in this study may have been too low to control the "rebound pain." There might also be another reason for why CISB could not control this "rebound pain." At the end of surgery, Iohexol (Omnipaque™, Cork, Ireland) was injected via the catheter to confirm the accurate location of the perineural catheter tip before commencing the continuous infusion of local anesthetics, and the contrast media might have acted as a barrier between the infused local anesthetics and brachial plexus. It was reported that contrast media can inhibit the effect of local anesthetics on account of its high viscosity,17) which reduces absorption in the perineural space. The position of the catheter tip was reconfirmed by fluoroscopy at the end of surgery due to possible catheter migration during the arthroscopic rotator cuff repair in the lateral decubitus position with 10 lb traction. However, displacement of the catheter did not occur, and it is believed that the radiographic reconfirmation using contrast media was unnecessary.

However, catheter retention was problematic in the patients in the CISB group. There was a 22% (7/32) incidence of accidental catheter retrieval in the CISB group, which is similar to that reported in other studies using the interscalene approach, i.e. 16% (4/25) and 12% (2/17) reported by Ilfeld et al.18) and Delaunay et al.,19) respectively. However, these incidences were higher than those reported by Sandefo et al.20) via the posterior approach, only 0.8% (1/122). Generally, mobilization exercises of the shoulder begin from one day after surgery for rotator cuff repair. Moreover, the shoulder patients were observed to have a wider range of postoperative activities than expected. Hence, maintenance of the CISB catheter around the neck might have certain problems.

One of the serious complications of CISB is neurological deficits of the upper extremities. A continuous peripheral nerve block appears to have a higher likelihood of neurological sequela due to the prolonged infusion of local anesthetics and the long period of perineural catheter indwelling. However, the incidence of permanent neurological complications was similar to that after a single interscalene block. Borgeat et al.14) reported minor neurological complications in 2.4%, 0.3%, and 0% of patients who received a continuous interscalene block at 1, 3, and 6 months postoperatively, respectively. Moreover, two (0.2%) out of 700 patients developed a sensory-motor deficit, which recovered at 19 and 28 weeks, respectively.2) In addition, according to Bishop et al.,1) minor complications were observed in 2.3% out of 512 patients who received a single interscalene block, and 0.2% had neuropathy at 6 months after surgery. Nevertheless, a continuous interscalene block carries the risk of neurological compromise in patients with preexisting neurological conditions.15) In the present study, all patients completely recovered from their motor weakness but one patient (4%) complained of residual sensory weakness or a tingling sensation for 3 weeks after surgery. According to these results and the literature, transient motor and sensory disturbances are often encountered after CISB, although complete neurological deficits are rare. However, most patients are quite uncomfortable and anxious about the prospect of such neurological complications, which might reduce the level of patient satisfaction concerning postoperative pain control. Nausea developed in two patients in CISB group. In contrast, there were no adverse systemic side effects encountered in the ISB-IB group. This suggests that ISB-IB is safer than CISB during the 48 hour infusion period in terms of preventing central nervous system toxicity.

The level of supplementary analgesic intake was similar in both groups. However, in the ISB-IB group, more analgesics were required 8 hours after surgery, which was 4 times that required at 1 hour after surgery. This was attributed to the 'rebound pain' after warning of the effect of the interscalene block. The same phenomenon was encountered during the previous study.3) Therefore, in cases that receive a single interscalene block, it would be more effective to give patients extra-analgesics during the early postoperative period to prevent "rebound pain." More study on ISB with an intra-bursal injection of ropivacaine administered in a PCA manner is needed.

There were some limitations in this study. As described above, the quantity and concentration of ropivacaine in both groups were different. Hence, a direct comparison was impossible. However, it is believed that this difference did not affect the overall results, i.e. ISB-IB is as effective as CISB with minimal complications at an analgesic concentration with prompt sensory recovery.

Second, all other possible methods of postoperative pain control were not included simultaneously. Nevertheless, a series of related studies regarding the efficient form of analgesia after rotator cuff repair were performed, and the next study will examine the appropriate method. Finally, bupivacaine is harmful to the cartilage, and many authors do not recommend its use in the glenohumeral space. Therefore, further study on the systemic or regional side-effects of ropivacaine is recommended.

In conclusion, a combination of a single interscalene block with a continuous intra-bursal infusion of ropivacaine may be an effective and safe alternative to a continuous interscalene block after arthroscopic rotator cuff repair, due to its similar analgesic effect and better catheter retention during infusion with a lower incidence of motor and sensory deficits.

## Figures and Tables

**Table 1:**
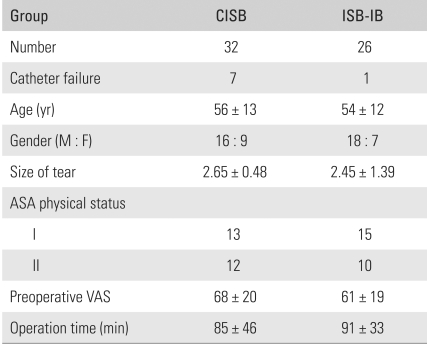
Demographic Data

CISB: A continuous interscalene block, ISB-IB: A single interscalene block combined with continuous intra-bursal infusion, ASA: American Society of Anesthesiologists, VAS: Visual analogue scale
